# Reduced temperature-dependent thermal conductivity of magnetite thin films by controlling film thickness

**DOI:** 10.1186/1556-276X-9-96

**Published:** 2014-02-26

**Authors:** No-Won Park, Won-Yong Lee, Jin-A Kim, Kyungjun Song, Hyuneui Lim, Wan-Doo Kim, Soon-Gil Yoon, Sang-Kwon Lee

**Affiliations:** 1 Department of Physics, Chung-Ang University, Seoul, 156–756, Republic of Korea; 2 Department of Materials Engineering, Chungnam National University, Daejeon 305-764, Republic of Korea; 3 Department of Nature-Inspired Nanoconvergence Systems, Korean Institute of Machinery and Materials (KIMM), Daejeon 305-343, Republic of Korea

**Keywords:** Iron oxide (Fe_3_O_4_), Thermal conductivity, 2D thin films, 3-*ω* technique, Callaway model, In-plane and out-of-plane

## Abstract

We report on the out-of-plane thermal conductivities of epitaxial Fe_3_O_4_ thin films with thicknesses of 100, 300, and 400 nm, prepared using pulsed laser deposition (PLD) on SiO_2_/Si substrates. The four-point probe three-omega (3-*ω*) method was used for thermal conductivity measurements of the Fe_3_O_4_ thin films in the temperature range of 20 to 300 K. By measuring the temperature-dependent thermal characteristics of the Fe_3_O_4_ thin films, we realized that their thermal conductivities significantly decreased with decreasing grain size and thickness of the films. The out-of-plane thermal conductivities of the Fe_3_O_4_ films were found to be in the range of 0.52 to 3.51 W/m · K at 300 K. For 100-nm film, we found that the thermal conductivity was as low as approximately 0.52 W/m · K, which was 1.7 to 11.5 order of magnitude lower than the thermal conductivity of bulk material at 300 K. Furthermore, we calculated the temperature dependence of the thermal conductivity of these Fe_3_O_4_ films using a simple theoretical Callaway model for comparison with the experimental data. We found that the Callaway model predictions agree reasonably with the experimental data. We then noticed that the thin film-based oxide materials could be efficient thermoelectric materials to achieve high performance in thermoelectric devices.

## Background

In recent decades, there has been a great interest in the application of thermoelectric (TE) effects in alternative clean energy sources [1-6]. For the evaluation of the thermoelectric performances of TE devices, their efficiencies can usually be quantified by a dimensionless figure of merit (*ZT*), *S*^2^*σT*/*κ* or a power factor *S*^2^*σ*, where *S* is the Seebeck coefficient, *σ* is the electrical conductivity, *κ* is the thermal conductivity, and *T* is the absolute temperature. High-performance thermoelectric materials with high *ZT* values should have a large Seebeck coefficient, high electrical conductivity, and low thermal conductivity [2,7,8]. To obtain an efficiently comparable to a household refrigerator, a *ZT* value at least 3 is desired for more widespread applications [6]. Recently, several researchers have alternatively studied two-dimensional (2D) thin films [9,10] to overcome the limitations of 1D nanostructured materials whose thermal properties are highly dependent on their dimensionality and morphology [3,11-13]. In 2010, Tang et al. reported that the thermal conductivity of holey Si thin film consistently reduces by around 2 orders of magnitude with a reduction in the pitch of the hexagonal holey pattern down to approximately 55 nm with approximately 35% porosity [9]. Similarly, Yu et al. reported that a Si nanomesh structure exhibits a substantially lower thermal conductivity than an equivalently prepared array of Si nanowires [10]. Hence, we believe that the 2D materials (i.e., thin film formation) could be highly promising candidates as TE materials for scalable and practical TE device applications.

Magnetite (Fe_3_O_4_) is a well-known half-metallic material, whose electronic density of states is 100% spin polarized at the Fermi level [14,15]. These properties allow Fe_3_O_4_ to be a promising candidate for spintronic devices [16]. However, the thermal property of this metal compound has not been widely studied. In 1962, Slack extensively studied and analyzed the thermal conductivity of a single crystal of paramagnetic bulk Fe_3_O_4_ materials at temperatures of 3 to 300 K [17]. He found that the thermal conductivity of Fe_3_O_4_ falls sharply with increasing temperature at the approximately 121 ± 2 K transition and reported a notable effect of vacancy and impurities on Fe_3_O_4_, particularly below 30 K. The thermal conductivity of pure Fe_3_O_4_ was as low as approximately 6 W/m · K at 300 K, owing to phonon scattering by local disorder in the materials, thus implying that pure Fe_3_O_4_ is a promising TE material. To the best of our knowledge, there have been no studies on the thermal properties of Fe_3_O_4_ thin films.

In this work, we present the out-of-plane thermal conductivities of epitaxial Fe_3_O_4_ thin films with thicknesses of 100 to 400 nm having different grain sizes and surface roughness. The films were grown at a deposition temperature of 300°C using pulsed laser deposition (PLD). We successfully demonstrated the temperature-dependent thermal conductivities of epitaxial Fe_3_O_4_ thin films via four-point probe 3-*ω* method in the temperature range of 20 to 300 K. The measured out-of-plane thermal conductivities of the Fe_3_O_4_ thin films (0.52 to 3.51 W/m · K) at 300 K are considerably reduced compared to those of the bulk materials (approximately 6 W/m · K) [17] because of strongly enhanced phonon-boundary scattering, as expected in the Callaway model [18]. Furthermore, we clearly realized that the thermal conductivity increased with an increase in film thickness and grain size, which agreed well with the theoretical predictions of the Callaway model.

## Methods

**Figure 1:**
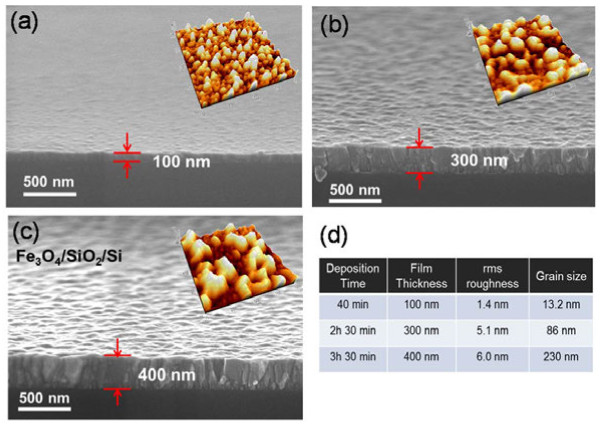
**SEM cross-sectional images of Fe**_**3**_**O**_**4 **_**thin films grown on a SiO**_**2**_**/Si substrate at 300°C using PLD. (a)** 100 nm, **(b)** 300 nm, and **(c)** 400 nm. The insets show the AFM images of each thin film. **(d)** A summary of the prepared Fe_3_O_4_ thin film, including rms roughness, film thickness, deposition time, and grain size information.

The epitaxial magnetite thin films were synthesized on SiO_2_/Si (100) substrates at a temperature of 300°C using PLD. The detailed growth processes can be found in our previous publication [19]. In brief, a krypton fluoride (KrF, 248 nm in wavelength) excimer laser whose energy density was approximately 2.1 J/cm^2^ at repetition rate of 4 Hz at a pressure of 10^-3^ Pa was used along with a ceramic target (pure, homogeneous, and highly dense α-Fe_2_O_3_ ceramic). Our previous results confirmed that the surface roughness of the films increased with increasing temperature. Consequently, the deposition temperature was maintained at 300°C to obtain a uniform quality in the grown films. The deposition rate of the films was maintained at approximately 1.2 nm/min. To measure the thermal conductivity, we prepared three Fe_3_O_4_ thin films with thicknesses of 100, 300, and 400 nm using PLD. X-ray diffraction confirmed that the films were grown with a (111) preferred orientation with high-quality epitaxial growth, as detected from the in-plane phi-scans of the films [19]. Figure 1a,b,c shows the cross-sectional scanning electron microscope (SEM) images of the as-grown Fe_3_O_4_ thin films, confirming that the thicknesses of the films were in the range of 100 to 400 nm. Atomic force microscope (AFM) images (insets of Figure 1ab,c) showed that the grown films exhibit smooth grain morphologies with a root-mean-square (rms) roughness of 1.4 to 6.0 nm, as summarized in Figure 1d. We also found that the grain size of the films increased from approximately 13.2 ± 5.2 nm to approximately 230 ± 23.10 nm when the film thickness was increased from 100 to 400 nm, indicating that thicker films have much rougher surface morphology and larger grain size.

**Figure 2:**
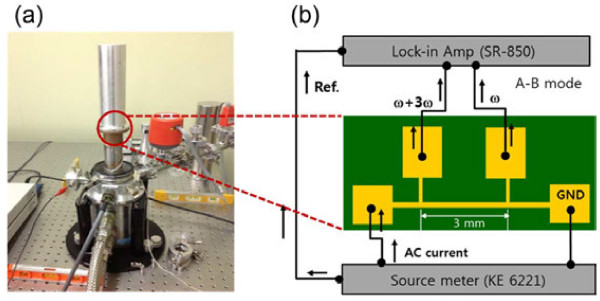
**Experimental setup including the circuit connections with thermal management and electrical measurement systems.** Experimental setup and circuit **(a)** and the corresponding circuit (right side) **(b)**, equipped with thermal management and electrical measurement systems for thermal conductivity measurements via the 3-*ω* method at temperature ranges of 20 to 300 K.

Prior to the thermal conductivity measurements, a 100-nm-thick SiO_2_ layer was deposited on the thin film through plasma-enhanced chemical vapor deposition for electrical insulation of the films. Finally, a narrow metal strip (Ti/Au = 10/300 nm) consisting of four-point probe electrodes acting as a heater wire and probe pads was patterned onto the specimen through a conventional photolithography process. The thermal transport measurements were performed in closed cycle refrigerator (CCR) system with a shielding box, as shown in Figure 2a, which helped maintain the temperature in the range of 20 to 300 K and provided a high-vacuum (approximately 10^-6^ Torr) environment to avoid heat loss. In the current study, we utilized a four-point probe 3-*ω* method based on the application of an alternating current (AC) with angular modulation frequency (1-*ω*), which was first developed by Cahill in 1990 [20] to measure the temperature-dependent thermal conductivities of as-grown Fe_3_O_4_ thin films. It has been proved the most promising technique to extract thermal conductivities of 1D nanostructures such as nanowires [21,22] and carbon nanotubes [23,24] and thin films [25-27]. We have also proved this technique to be one of the powerful methods to extract the thermal conductivity of most low-dimensional materials [21]. Our experimental setup reported previously [21] is similar to the original design by Cahill [20] and adheres to the experimental design by Feser et al*.*[25].

Figure 2a,b shows the experimental setup including the circuit connections with thermal management and electrical measurement systems for out-of-plane thermal conductivity measurements via the 3-*ω* method. In brief, the sample was first attached to a printed circuit board substrate with vacuum grease for mounting inside a CCR with a shielding box. The source meter (Keithley 6221, Cleveland, OH, USA) was connected to both metallic pads to generate an AC (*I*_0_), as shown in Figure 2b. *I*_0_ with an angular modulation frequency of 1-*ω* was applied to generate Joule heat and temperature fluctuations at a frequency of 2-*ω*. The resistance of the narrow metal strip is proportional to the temperature that leads to a voltage fluctuation (*V = IR*) of 3-*ω* across the specimen. A lock-in amplifier (A-B mode, SR-850, Stanford Research System, Sunnyvale, CA, USA) connected to the two electrodes in the middle received the 3-*ω* voltage fluctuation along the narrow metal strip; this gives the information on the thermal conductivity of the films (as indicated in Figure 2b). To measure the thermal conductivity of the thin films, we then plotted the third-harmonic voltage (*V*_3*ω*_) against the natural logarithm of the applied frequencies (ln *ω*), which showed a linear relationship. Consequently, we determined the out-of-plane thermal conductivity from the slope in the linear region. The difference between two *V*_3*ω*_ values (i.e., *V*_3*ω*1_ and *V*_3*ω*2_) is equated to the temperature drop across the Fe_3_O_4_ film and is used to calculate the cross-plane thermal conductivity, which is defined by the following equation:

(1) $$\kappa =\frac{V_{0}^{3}\cdot \ln\left(\frac{\omega_{2}}{\omega_{1}}\right)}{4\pi lR_{0}^{2}\left(V_{3\omega_{1}}-V_{3\omega_{2}}\right)}\frac{\mathrm{dR}}{\mathrm{dT}}$$

**Figure 3:**
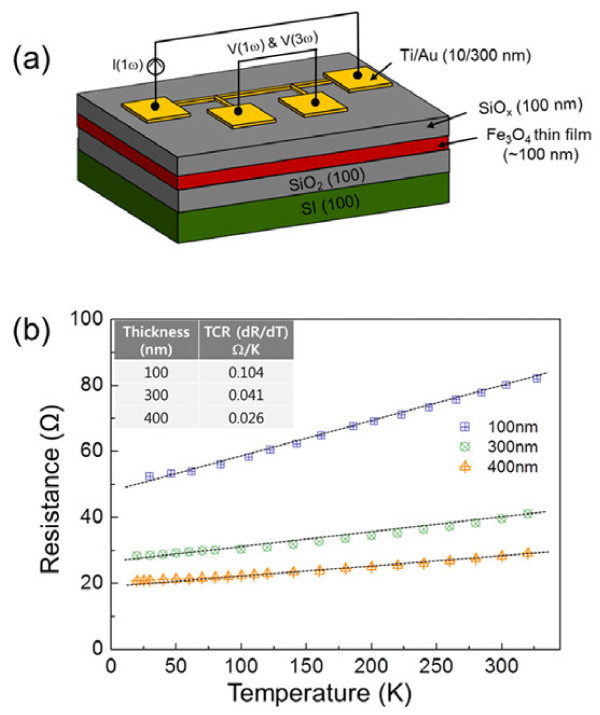
**Four-point probe 3-*ω *method and temperature-dependent resistances. (a)** Schematic view of the four-point probe 3-*ω* method where the out-of-plane thermal conductivity can be measured. **(b)** The temperature-dependent resistances of three Fe_3_O_4_ thin films (100, 300, 400 nm in thickness) at temperature ranges of 20 to 300 K.

Here, *V*_0_ and *R*_0_ are the applied voltage and electrical resistance, respectively, along the heater wire of length *l*. $\;V_{3\omega_{1}}$ and $V_{3\omega_{2}}$ are the third-harmonic voltages at input current frequencies of *ω*_1_ and *ω*_2_, respectively, and *dR/dT* (temperature coefficient resistance, TCR) is the rate of the resistance change of the heater at temperatures of 20 to 300 K. Figure 3a shows a schematic of the four-point probe electrodes patterned onto SiO_*x*_/Fe_3_O_4_/SiO_2_/Si substrate for thermal conductivity measurements using the 3-*ω* method. To confirm our results of thermal conductivity measured using the four-point probe 3-*ω* method, we used bismuth (Bi) films (50 nm in thickness) whose thermal conductivity is well known, as a reference sample. We determined its thermal conductivity to be 2.7 to 2.9 W/m · K, which is in good agreement with the previous reported results by Völklein and Kessler [28] and Völklein et al. [29] who reported that the thermal conductivity of 60-nm Bi thin films was approximately 3.6 W/m · K at 300 K. Thus, our experimental setup and the associated analysis via the four-point probe 3-*ω* method were clearly validated through a comparison with the results for reference sample. Figure 3b shows temperature-dependent resistances of the three Fe_3_O_4_ thin films (100, 300, 400 nm in thickness) in the temperature range of 20 to 300 K. The relationship between the resistance changes in the heater wire and the temperature is linear. Figure 3b shows that the TCR for the 100-, 300-, and 400-nm Fe_3_O_4_ thin films is approximately 0.104 Ω/K, approximately 0.041 Ω/K, and approximately 0.026 Ω/K, respectively. These values can be used for estimating thermal conductivity as defined in Equation 1.

## Results and discussion

**Figure 4:**
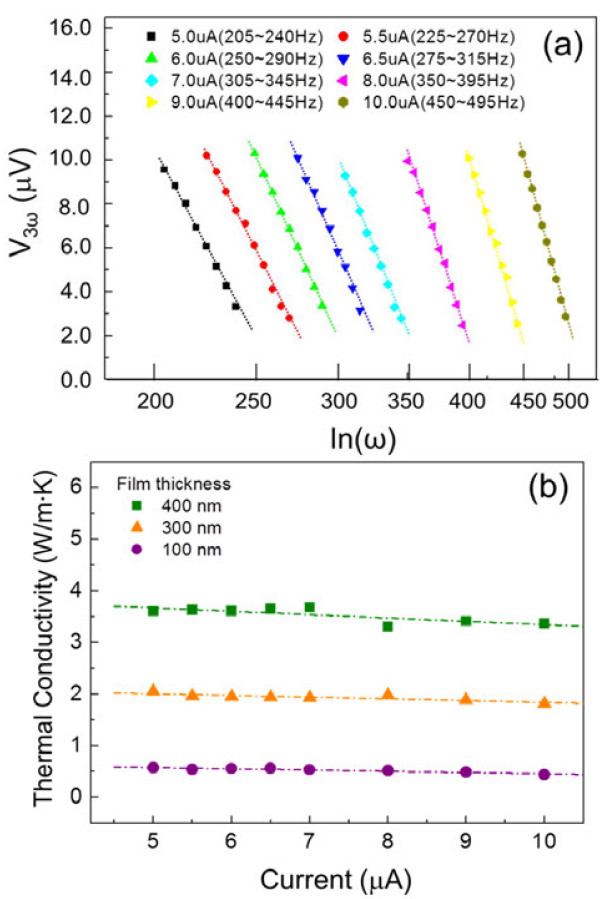
***V***_**3*ω***_**distribution and thermal conductivities of the Fe**_**3**_**O**_**4 **_**film. (a)** Linear regions of the third-harmonic voltage versus the applied frequency at various applied alternating currents (AC) ranging from 5 to 10 μA. **(b)** Thermal conductivities of Fe_3_O_4_ film with different film thicknesses (100, 300, and 400 nm) with respect to the applied AC (5 to 10 μA). Variation in the thermal conductivity with modulation of the input AC current could be assumed as measurement errors in thermal conductivity.

To ensure that the measured *V*_3*ω*_ signal is generated by the Fe_3_O_4_ thin film, we investigated the variation in the signal with the applied frequency (ln *ω*) from the 3-*ω* measurements. This applied frequency usually provides a suitable current range for an estimation of the *V*_3*ω*_ signal from the sample. As discussed previously by Cahill [20], the linear relationship of ln *ω* with *V*_3*ω*_ should be satisfied as shown in Figure 4a. Figure 4a presents the *V*_3*ω*_ distribution of the 100-nm Fe_3_O_4_ thin film for different applied frequencies. The *V*_3*ω*_ signal follows ln *ω* dependence very well in the frequency range of 205 to 495 Hz, which agrees well with Equation 1. To validate the measured *V*_3*ω*_ signal and the thermal conductivity (*κ*) from the 3-ω measurements, we studied the applied current dependence on the thermal conductivity by applying an AC of 5 to 10 μA. As shown in Figure 4b, the measured thermal conductivities of the films with thicknesses of 100, 300, and 400 nm were approximately 0.52 ± 0.05, approximately 1.92 ± 0.06, and approximately 3.51 ± 0.12 W/m · K, respectively, in the applied current range, indicating that *κ* is independent of the applied current (*I*_0_). We found that the errors in the thermal conductivity measurements are less than approximately 3% to 9%, depending on the film thickness.

**Figure 5:**
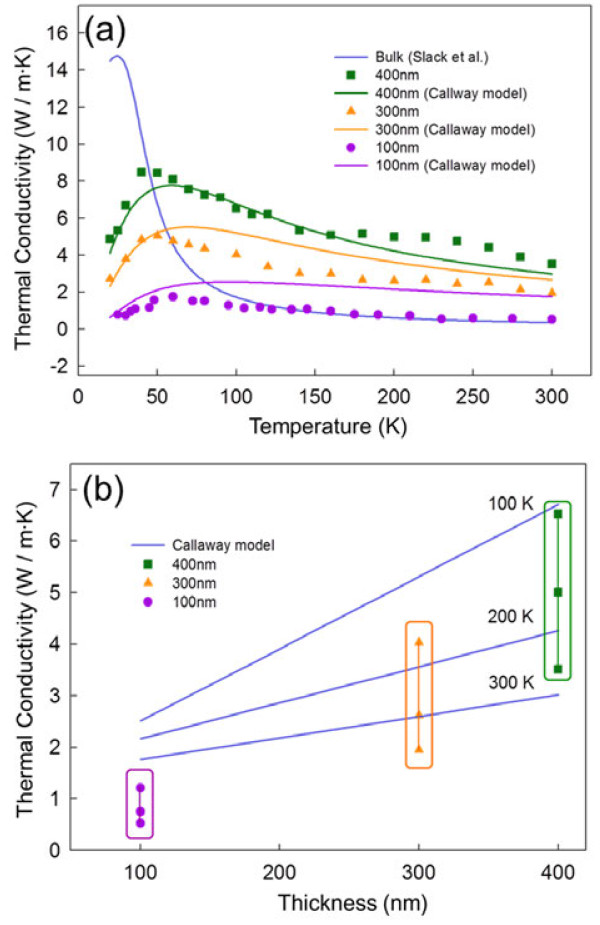
**Temperature-dependent conductivities of three Fe**_**3**_**O**_**4 **_**films and a simple theoretical calculation based on the Callaway model. (a**, **b)** Measured thermal conductivities of 100-, 300-, and 400-nm-thick Fe_3_O_4_ thin films at temperatures of 20 to 300 K using the 3-*ω* method, including the thermal conductivity of bulk materials. The solid line denotes thermal conductivity of bulk materials from the theoretical Callaway model, which includes the effect of the impurity, Umklapp process, boundary scattering with film grain size, and film thickness.

Figure 5a shows the temperature dependence of out-of-plane thermal conductivity of three Fe_3_O_4_ films at temperatures of 20 to 300 K and a simple theoretical calculation based on the Callaway model (solid lines in the figure) to compare with the experimental results (discussed in the next section). For the 400-nm-thick films, the thermal conductivity increased with increasing temperature up to approximately 40 K, then decreased with increasing temperature up to 300 K. Similar behaviors were observed for the other thin films (100 and 300 nm), as shown in Figure 5a. The phonon-phonon Umklapp and phonon-boundary scattering play an important role in phonon transport, and thus, the thermal conductivity decreases with increasing temperature [30,31]. Thus, we characterized the peaks of thermal conductivity (Umklapp peak) for the thin films whose thicknesses were 100, 300, and 400 nm, respectively. Our results presented in Figure 5a show that with the decrease in the film thickness from 400 to 100 nm, the corresponding Umklapp peaks shifted by approximately 20 K. According to the previous work in bulk F_3_O_4_, the Umklapp peak was generally observed at approximately 30 K [17], which is much lower than that for the thin films (approximately 40 to 60 K as shown in Figure 5a). From the shift in the Umklapp peaks, we can also confirm that phonon-boundary scattering is clearly dominant in the films in the temperature range of 40 to 60 K as a result of the grain size and film thicknesses [32,33]. In addition, when the temperature is above 50 K, the phonon-phonon Umklapp scattering becomes more pronounced. Our observation was in good agreement with a previous report on the thermal conductivity of 1D Bi nanowires [21]. Figure 5b presents the dependence of out-of-plane thermal conductivity on the film thickness at temperatures of 100, 200, and 300 K. The values of κ for the corresponding film thicknesses 100, 300, and 400 nm at 300 K increased gradually to approximately 0.52, approximately 1.85, and approximately 3.51 W/m · K, respectively. We also found that the thermal conductivities of the films were 1.7 to 11.5 times lower than that of bulk Fe_3_O_4_ (approximately 6 W/m · K) [17]. It has been well understood that the significant reduction in the thermal conductivity of the thin films (100 to 400 nm in thickness) compared to the bulk materials could be due to the enhanced phonon-boundary scattering in thin films predicted previously by Callaway [18]. In addition, we added the theoretical calculation results of Callaway's model in the same figure (solid line in Figure 5a,b). The results predicted by the Callaway model agree reasonably well with the experimental data, including the results for bulk Fe_3_O_4_. We can thus confirm that the significant reduction in the thermal conductivity for nanoscale thin films is principally a result of phonon-boundary scattering. In the following section, the calculation model is discussed in detail.

To determine the temperature dependence of the thermal conductivity, *κ*(*T*), in Fe_3_O_4_ thin films quantitatively, we performed a theoretical calculation (i.e., fitting) based on the relaxation time model using the following expression predicted by Callaway in 1959 [18]:

(2) $$\kappa \left(T\right)=\frac{k_{B}}{2\pi^{2}c}{\left(\frac{k_{B}T}{\hbar }\right)}^{3}\;{\int_{0}^{\theta }}^{D/T}\tau_{c}\frac{x^{4}e^{x}}{{\left(e^{x}-1\right)}^{2}}\mathrm{dx},$$

where *ω* is the phonon frequency, *k*_B_ is the Boltzman constant, *ℏ* is the reduced Planck constant, *x* denotes the dimensionless parameter, *x* = *ℏω*/*k*_B_*T*, *θ*_D_ is the Debye temperature, *T* is the absolute temperature, and *c* is the velocity of sound. The total combined phonon scattering rate (relaxation time, *τ*_c_) is given by

(3) $$\tau_{c}^{-1}=\frac{c}{d_{1}}+A\omega^{4}+B\omega^{2}Te^{\left(-\frac{\theta_{D}}{3T}\right)},$$

where *d*_1_ is the grain size of the thin films (approximately 13.2, approximately 86, approximately 230 nm for the 100-, 300-, and 400-nm-thick films, respectively, from the AFM measurements shown in Figure 1), *A* and *B* are independent parameters of temperature and fitting, respectively, and *c* is the sound velocity, which is highly dependent on the direction of movement of phonons (average *c* = 2,500 m/s) [17]. To add the film thickness in Equation 3, we modified the phonon scattering rate given as

(4) $$\tau_{c}^{-1}=c\left(\frac{1}{d_{1}}+\frac{1}{d_{2}}\right)+A\omega^{4}+B\omega^{2}Te^{\left(-\frac{\theta_{D}}{3T}\right)},$$

where *d*_2_ is the corresponding film thickness. For the Fe_3_O_4_ films, we estimated that the values of *A* and *B* in Equation 4 were numerically optimized as approximately 8.46 × 10^-43^ *S*^3^ and approximately 7.89 × 10^-18^ *S*/*K*, respectively, from the fitting to the bulk material values [17]. According to the Callaway model in Equations 3 and 4, the first term $c\left(\frac{1}{d_{1}}+\frac{1}{d_{2}}\right)$ represents the boundary scattering; the second term *A*ω^4^ represents the scattering by point impurities or isotopes, and the third term $B\omega^{2}Te^{\left(-\frac{\theta_{D}}{3T}\right)}$ represents the Umklapp process. Theoretical fits of the temperature dependence of the out-of-plane thermal conductivities of the Fe_3_O_4_ films from 20 to 300 K of Equations 2 and 4, which were obtained using the commercial application Mathematica (http://www.wolfram.com), are compared with the experimental results in Figure 5a,b. From the numerical calculation of the temperature dependence of thermal conductivity, it was noted that the *κ* values indisputably decreased when the grain size was reduced, indicating that the effect of the nano-grained thin films on the thermal conductivity is essentially due to the relaxation time model based on phonon-boundary scattering. As shown in Figure 5a,b, the theoretical modeling based on the Callaway model agrees well quantitatively with the experimental data even though there is a difference in the *κ* values between the theoretical and experimental results for the 100-nm Fe_3_O_4_ film. The measured thermal conductivity results in the 100-nm films were approximately five times lower than the Callaway model prediction. This deviation can be explained by two arguments. First, the deviation in the thermal conductivity for the 100-nm thick film could be explained by the boundary effect, i.e., surface boundary scattering of the thinner films, in which the surface boundary scattering is more dominant compared to that of bulk and bulk-like thicker films, providing more phonon-boundary effect in thermal conductivity. However, in our theoretical model, no size and surface boundary scattering effects were considered. Thus, the measured temperature dependence of the thermal conductivity (0.52 W/m · K at 300 K) was relatively lower than the results expected from the theoretical calculation (1.9 to 2.4 W/m · K at 300 K), as shown in Figure 5b [2,34,35]. Previously, Li et al. also reported a similar observation for the thermal conductivity of Bi_2_Se_3_ nanoribbon [36]. Second, to numerically calculate the thermal conductivity using the Callaway model, we used the fitting parameters of *A* and *B* in the relaxation rate from the bulk materials. Thus, the theoretical calculation could be closer to the bulk material values. To clearly understand this inconsistency between the theoretical and experimental results, especially in nanoscale thin films (100-nm thin film in our case), the size and surface boundary effects in the Callaway model should be studied in detail for 1D and 2D nanostructures.

**Figure 6:**
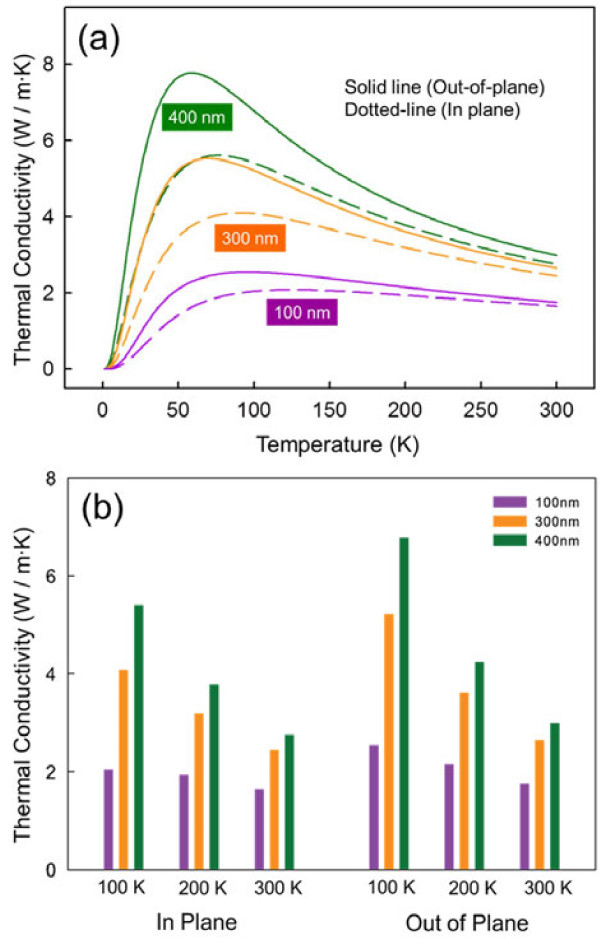
**Thermal conductivities of 100-, 300-, and 400-nm-thick Fe**_**3**_**O**_**4 **_**thin films. (a, b)** Calculated thermal conductivities of 100-, 300-, and 400-nm-thick Fe_3_O_4_ thin films at temperatures of 20 to 300 K obtained using the simple Callaway phonon scattering model. The temperature-dependent in-plane thermal conductivity was calculated by modifying the Debye temperature and sound velocity in the Callaway phonon scattering model.

To measure the in-plane thermal conductivity of the nanoscale thin films from the out-of-plane thermal conductivity, we re-analyzed the theoretical Callaway model with the longitudinal component of sound velocity and Debye temperature, which were highly dependent on the direction of movement of phonons. In this calculation, we assumed that our measured results are close to the theoretical prediction, as shown in Figure 5a,b. The average sound velocity can be decomposed into the transverse and longitudinal components as defined in [37]:

(5) $$\frac{3}{c^{3}}=\frac{1}{c_{L}^{3}}+\frac{2}{c_{T}^{3}},$$

where *c*_T_ (approximately 2,305.4 m/s) and *c*_L_ (approximately 3,263.3 m/s) are the transverse and longitudinal velocities, respectively. In addition, the Debye temperature depends on sound velocity. Thus, using the calculated *c*_T_ in Equation 5, we could calculate the Debye temperature for transverse component ($\theta_{D}^{T}$) given as

(6) $$\;\theta_{D}^{T}={\left(\frac{6\pi^{2}}{V}\right)}^{\frac{1}{3}}\frac{\hbar c_{T}}{k_{B}},$$

where *V* is the volume per atom (approximately 10.54 × 10^-30^ m^3^). The Debye temperature with transverse sound velocity is then determined to be approximately 313.1 K. Finally, we calculated the in-plane thermal conductivity of the Fe_3_O_4_ films with transverse components of sound velocity (*c*_T_) and Debye temperature ($\theta_{D}^{T}$) using Equation 2. Figure 6a,b shows calculated both in-plane and out-of-plane thermal conductivities of 100-, 300-, and 400-nm-thick Fe_3_O_4_ thin films at temperatures of 20 to 300 K obtained using the simple Callaway phonon scattering model. As shown in Figure 6a, the deviation in thermal conductivity between the out-of-plane and in-plane thermal conductivities decreased with increasing temperature. At room temperature, the out-of-plane and in-plane thermal conductivities were determined to be 1.7 to 3.0 and 1.6 to 2.8 W/m · K, respectively. It was also noticed that the calculated out-of-plane thermal conductivity values are slightly higher than the in-plane thermal conductivity values in the Fe_3_O_4_ thin film as shown in Figure 6. This behavior could be due to the columnar structures of the grains (see Figure 1), where the phonons moving transversally in the Fe_3_O_4_ films are scattered by the columnar grains in the films. Similar results can be seen in diamond thin film grown by chemical vapor deposition (CVD), where the measured out-of-plane thermal conductivity consistently show a higher thermal conductivity along the columnar grains than the in-plane thermal conductivity [38].

## Conclusion

In summary, we first present the thermal conductivity of epitaxial Fe_3_O_4_ thin films with thicknesses of 100 to 400 nm prepared on SiO_2_/Si (100) substrates using PLD. By measuring the temperature-dependent thermal characteristics of three Fe_3_O_4_ thin films using the effective four-point probe 3-*ω* method, we found that the thermal conductivities of the films are greatly reduced when compared with those of the corresponding bulk materials and that the thermal conductivity decreases with decreasing film thickness from 400 to 100 nm. Both theoretical and experimental results indicate that the Umklapp peaks of the thermal conductivity of Fe_3_O_4_ films move toward higher temperatures with decreasing film thickness, owing to the phonon-boundary scattering. The thermal conductivity was found to be in the range of 0.52 to 3.51 W/m · K at 300 K, which was 1.7 to 11.5 orders of magnitude lower than that of bulk materials at 300 K. We found that the modified Callaway theoretical model including the film thickness effect agreed well with the results obtained using the 3-*ω* method. Furthermore, we indirectly measured the in-plane thermal conductivity by re-analyzing the Callaway model using the measured out-of-plane thermal conductivity. We then suggested that the thin film-based oxide materials could be promising candidates as thermoelectric materials to achieve high-performance TE devices.

## Competing interests

The authors declare that they have no competing interests.

## Authors' contributions

NWP and WYL, and JAK carried out all the experiments and analysis including the sample growth. KS, HEL, SGY, and WDK helped discuss the sample analysis and provided part of the financial support. SKL organized the final manuscript. All authors read and approved the final manuscript.
